# Recent trends in the applications of thermally expanded graphite for energy storage and sensors – a review

**DOI:** 10.1039/d1na00109d

**Published:** 2021-09-16

**Authors:** Preethika Murugan, Ramila D. Nagarajan, Brahmari H. Shetty, Mani Govindasamy, Ashok K. Sundramoorthy

**Affiliations:** Department of Chemistry, SRM Institute of Science and Technology Kattankulathur 603 203 Tamil Nadu India ashokkus@srmist.edu.in; Department of Materials Science and Engineering, National Taipei University of Technology (Taipei Tech) Taiwan; Department of Physics & Nanotechnology, SRM Institute of Science and Technology Kattankulathur 603 203 Tamil Nadu India

## Abstract

Carbon nanomaterials such as carbon dots (0D), carbon nanotubes (1D), graphene (2D), and graphite (3D) have been exploited as electrode materials for various applications because of their high active surface area, thermal conductivity, high chemical stability and easy availability. In addition, due to the strong affinity between carbon nanomaterials and various catalysts, they can easily form metal carbides (examples: ionic, covalent, interstitial and intermediate transition metal carbides) and also help in the stable dispersion of catalysts on the surface of carbon nanomaterials. Thermally expanded graphite (TEG) is a vermicular-structured carbon material that can be prepared by heating expandable graphite up to 1150 °C using a muffle or tubular furnace. At high temperatures, the thermal expansion of graphite occurred by the intercalation of ions (examples: SO_4_^2−^, NO_3_^−^, Li^+^, Na^+^, K^+^, *etc.*) and oxidizing agents (examples: ammonium persulfate, H_2_O_2_, potassium nitrate, potassium dichromate, potassium permanganate, *etc.*) which helped in the exfoliation process. Finally, the obtained TEG, an intumescent form of graphite, has been used in the preparation of composite materials with various conducting polymers (examples: epoxy, poly(styrene-*co*-acrylonitrile), polyaniline, *etc.*) and metal chlorides (examples: FeCl_3_, CuCl_2_, and ZnCl_2_) for hydrogen storage, thermal energy storage, fuel cells, batteries, supercapacitors, sensors, *etc.* The main features of TEG include a highly porous structure, very lightweight with an apparent density (0.002–0.02 g cm^−3^), high mechanical properties (10 MPa), thermal conductivity (25–470 W m^−1^ K^−1^), high electrical conductivity (106–108 S cm^−1^) and low-cost. The porosity and expansion ratio of graphite layers could be customized by controlling the temperature and selection of intercalation ions according to the demand. Recently, TEG based composites prepared with metal oxides, chlorides and polymers have been demonstrated for their use in energy production, energy storage, and electrochemical (bio-) sensors (examples: urea, organic pollutants, Cd^2+^, Pb^2+^, *etc.*). In this review, we have highlighted and summarized the recent developments in TEG-based composites and their potential applications in energy storage, fuel cells and sensors with hand-picked examples.

## Abstract 2

This review highlight and summarizes the latest developments on the synthesis of TEG based composite materials for their applications in hydrogen storage, thermal energy storage, fuel cells, batteries, supercapacitors, and (bio-) sensors.

## Graphical abstract

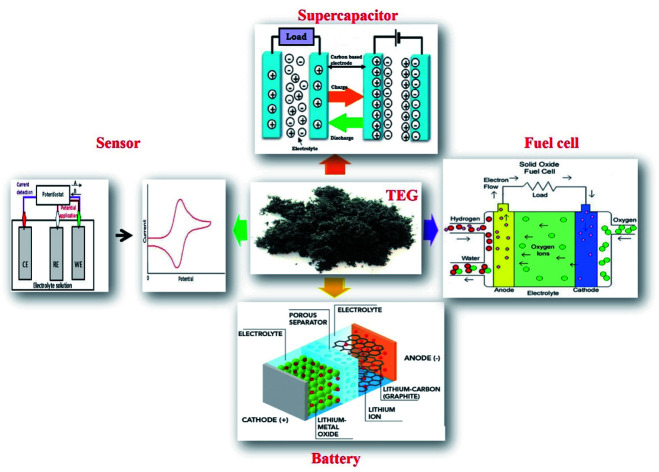

## 1. Introduction

Generally, various electrode materials used in fuel cells,^1^ batteries,^2^ supercapacitors,^3^ and electrochemical sensors^4^ may suffer from specific problems such as poor mass transport, easy contamination of the catalyst surface, poor thermal and electrochemical stabilities, loss of activity with time, *etc.*^5^ In order to increase the performance of the devices with longer life, the electrode materials should have high mass transport, easily renewable catalyst surface, high thermal and electrochemical stabilities, high conductivity, high corrosion resistance and good mechanical properties.^5^ It is apparent that the performance of the electrode materials could be improved by the addition of functional additives^6^ such as activated carbon, carbon black (CB),^7^ carbon nanodots,^8^ carbon nanotubes (CNTs),^9^ graphene^10^ and graphite nanoparticles^11^ which all enhanced the electrochemical activity of the electrode materials.^12,13^ For example, Glebova *et al.* demonstrated that thermally expanded graphite (TEG) can improve the thermal stability of the Nafion membrane for use in fuel cells.^5^ Due to the perfect crystalline structure, carbon materials have been synthesized in various dimensions (examples: zero-dimensional (0D), one-dimensional (1D), two-dimensional (2D) and three-dimensional (3D)) to improve the electrochemical activity of the composite materials.^14^ For comparison purposes, the surface area, electrical and thermal conductivities of CNTs, graphite, TEG, graphene and CB are shown in Table 1. As a promising choice, carbon nanomaterials (0D: carbon dots,^15,16^ fullerenes^17^ and nanodiamond;^18^ 1D: single and multi-walled CNTs,^19^ carbon nanohorns^20^ and graphene nanoribbons;^21^ 2D: graphene,^22^ graphene oxide (GO)^23^ and 3D: graphite,^24^ TEG,^25^*etc.*) have been used to provide high chemical stability, large surface-active area, good flexibility, thermal conductivity, high electrical conductivity, long-term stability and environmental friendliness with definite geometry.^26^ Therefore, the above mentioned carbon materials have been successfully demonstrated in various electrochemical applications.^27–30^ In addition, due to the good affinity between carbon materials and metal catalysts, carbon materials were able to form metal-carbides and uniform dispersion of metal catalysts on the surface of the electrode.^31^ Moreover, the usage of noble metal-based catalysts can be reduced by the addition of carbon materials to some extent for cost reduction without affecting the efficiency of the electrode. Generally, carbon black, CNTs, carbon nanofibers and graphene were predominately used in energy-related applications.^32^ CNT based composite materials showed excellent conductivity and high thermal and mechanical properties.^33–35^ But, the usage of CNTs in manufacturing commercial products has still been limited due to some of the existing challenges such as high production cost, limited production capacity, safety, less purity, *etc.*^36–38^ As a potential alternative, graphite is readily available at low cost^39^ with high corrosion resistance, high thermal and electrical conductivities^40^ (Table 1). It can be used as a functional additive in various applications.^41,42^

**Table 1:** Comparison of the surface area and electrical and thermal properties of various carbon materials

S. no.	Carbon materials	Dimension of the materials	Active surface area (m^2^ g^−1^)	Thermal conductivity (W m^−1^ K^−1^)	Electrical conductivity (S cm^−1^)	Bulk density (g cm^−3^)	References
1	Graphite	3D	3.5–20	100–400	10^5^	1–1.8	43 and 44
2	TEG	3D	40–200	25–470	10^6^ to 10^8^	0.007	45 and 46
3	CB	3D	50–100	6–174	10^3^	0.9–2.1	47 and 48
4	CNTs	1D	120–500	2000–6000	10^2^ to 10^6^	0.14–0.28	49 and 50
5	Graphene	2D	180	5300	10^7^ to 10^8^	0.002–0.2	47 and 51

**Fig. 1:**
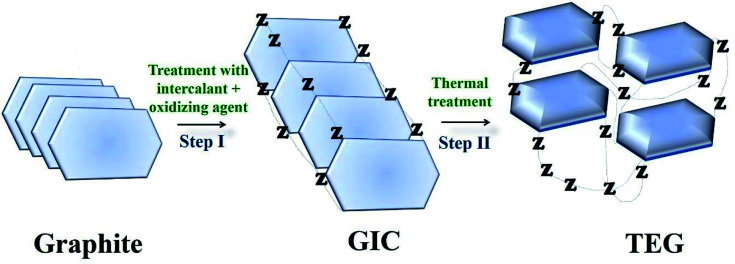
Schematic representation of TEG synthesis. Z − represents the intercalating ions (examples: SO_4_^2−^, NO_3_^−^, formic acid, metals, metal chlorides, oxides and halogens) which occupied the space in between the graphite layers.^59^

**Fig. 2:**
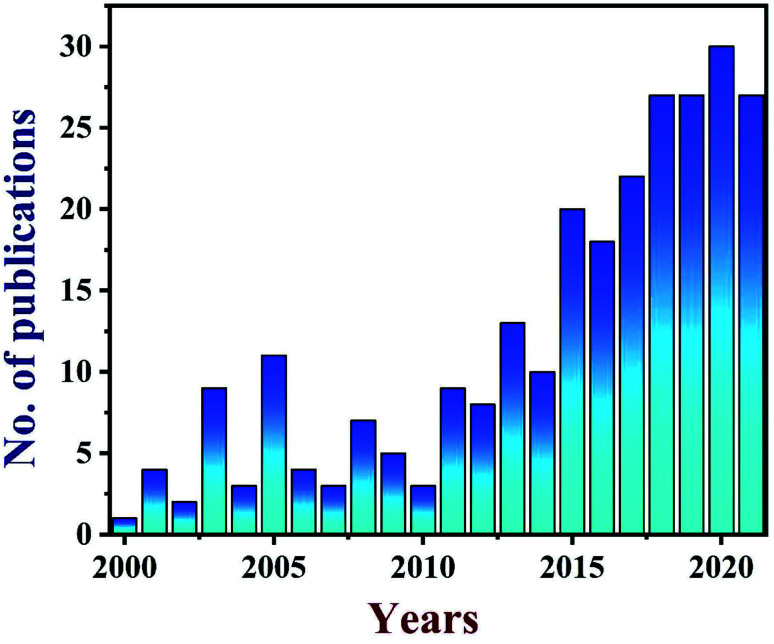
Bar graph showing the increasing trend in the number of publications on “TEG” from 2000 to July 2021. These data were obtained using the keyword “thermally expanded graphite” from the Scopus database.

TEG is a vermicular or a worm-like structured non-toxic layered material which exhibits good flexibility, high chemical tolerance and excellent thermal shock resistance.^52–54^ TEG (a multi-porous (2–10 nm) material) was synthesized by treating graphite^55,56^ with various ions and compounds (examples: sulphate (SO_4_^2−^), nitrate (NO_3_^−^), formic acid, aluminum chloride, ferric chloride (FeCl_3_), halogens, alkali metals, *etc.*) under constant stirring at room temperature for a few hours (Fig. 1, stage 1). This treated graphite was also known as the graphite intercalation compound (GIC).^57,58^ In the second step, the GIC was thermally heated from 300–1150 °C to obtain TEG (Fig. 1, stage 2). So far, the total number of publications reported on TEG was estimated using the keyword “thermally expanded graphite” from the Scopus database (Fig. 2). It is evident that the number of publications on TEG based materials are increasing.

**Fig. 3:**
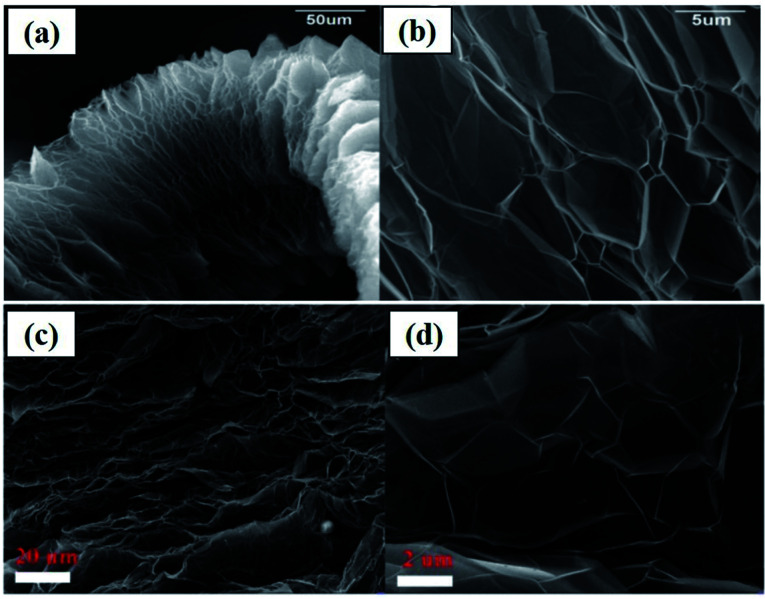
(a) Scanning electron microscopy (SEM) images of TEG which exhibited a worm-like-morphology, (b) bulk expansion along the crystallographic axis of graphite which is larger than its original size.^40^ (c) and (d) SEM images of the layered structure of TEG.^60^ These images were adapted with permission from ref. 40 and 60 copyright 2020 Elsevier.

As an example, the expanded layers of TEG are shown in Fig. 3(a–d). Even after the thermal expansion process, graphite layers were attached to each other by van der Waals forces of attraction.^40,60^ Finally, the obtained TEG was used as a fire retardant,^61^ high-performance gasket,^62^ conductive filler,^63^ host material for catalyst loading,^64^ phase-changing material,^65,66^ radiation shield,^67,68^ electrode material,^69,70^ and foundry product.^71^

### 1.1 Intercalation process of graphite

The process of intercalation involves the insertion of ions between the layers of bulk graphite. Various chemical compounds have been used as intercalates to synthesize TEG. For example, SO_4_^2−^,^72^ NO_3_^−^,^73^ organic acids,^74^ aluminum chloride,^75^ FeCl_3_,^76^ halogens,^77^ alkali metals,^78^ other metal halides,^79^*etc.* have been employed as intercalates to synthesise TEG. Among these, SO_4_^2−^ have been selectively used to prepare GICs such as “graphite bisulfate”.^56^

**Fig. 4:**
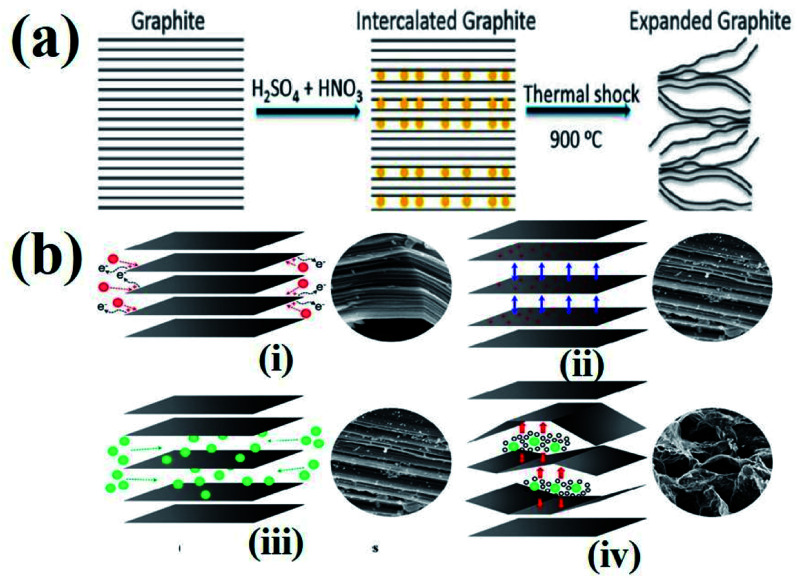
(a) Schematic representation of the TEG expansion process in the presence of intercalates between the adjacent layers of graphene.^83^ (b) Step by step synthesis process of TEG: (i) peroxidation, (ii) opening of the layers, (iii) penetration of intercalants and (iv) expansion at high temperature.^84^ These images were adapted with permission from ref. 83 and 84 copyright 2020 Elsevier and MDPI.

However, during the synthesis of TEG by acid treatment with metal intercalates, various functional groups (–OH, –COOH, *etc.*) were also introduced on the TEG surface by the oxidation process. In this way, the obtained TEG exhibited enhanced properties due to the presence of intercalates in between the graphite layers.^41^ For example, compared to natural graphite flakes, sulfate intercalated TEG showed an increase in the active surface area from 1.5 to 45.5 m^2^ g^−1^.^80^ It is worth noting that during thermal heating of acid treated graphite, intercalates were converted from a solid to a gaseous phase^81^ and the gas formation helped in the expansion of graphite layers (an increase in volume was observed). It was also suggested that the pressure generated in between the layers might have increased the distance between adjacent layers to move apart,^82^ as shown in Fig. 4(a) and (b). Depending on the intercalation process, various functional groups (amine, imine, carboxyl, hydroxyl, epoxide, and lactone) could be formed on the GIC, which was beneficial for establishing a good interface. Later, the presence of these functional groups could bring lattice defects on the expanded TEG and inversely affect the electrical and thermal conductivities of the nanofiller.

### 1.2 Effects of the intercalation process on graphite

Generally, the applied temperature and mesh size of the graphite could affect the expansion ratio of the graphite flakes.^85^ As said earlier, TEG could be synthesized by supplying heat in the presence of selected intercalant compounds. The expansion of layers resulted in crystallographic de-lamination that occurred in parallel to the “*c*” crystallographic axis of the graphite flakes.^86^ Elevated temperature causes the expansion agent to vaporize (as CO_2_, SO_2_, and H_2_O) by producing enough pressure to push out the adjacent graphite layers away from each other. As a consequence of the expansion process, a decrease in the bulk density and a 10-fold increase in the surface area were observed for TEG with the enhanced physical and chemical properties.^87^ For example, the surface area, density, crystallographic structure, electronic behavior, chemical reactivity, and intumescent nature of TEG have been significantly improved.^88^

The maximum rate of expansion was observed for a specific grade of graphite when exposed at high temperatures in a pre-heated furnace between 600 and 1200 °C.^89^ It should be noted that slow heating during the thermal expansion process did not help to expand graphite layers as required because the process of graphite expansion was mainly dependent on the extreme forces created by a rapid gas explosion.^90^ In addition, the mesh size of the graphite was directly proportional to the expansion ratio of TEG. For example, the larger graphite flakes (10 mesh) showed a higher expansion ratio than smaller flakes (325 mesh). This was due to the fast escape of the gas molecules (CO_2_, SO_2_, and H_2_O) and they failed to create the required pressure to expand the adjacent graphene layers.^89^ In the case of larger flakes, gas molecules could help to expand the layers away from each other. Similarly, the thicker flakes can expand more proportionally than the thinner flakes.^91^ On the other hand, the TEG expansion process could generate more acid wastes and the rate of expansion was uncontrollable during the synthesis process. Also, a high temperature was necessary to achieve the best expansion of TEG with high product yield.

## 2. Preparation of TEG

**Scheme 1:**
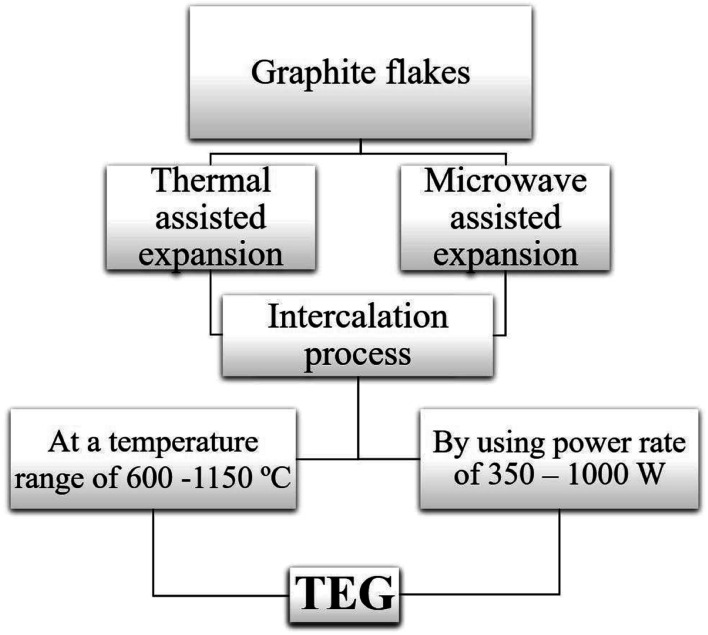
Flowchart representation of the synthesis of TEG from graphite.

Two common methods such as (i) thermal assisted expansion and (ii) microwave assisted expansion were reported for the synthesis of TEG (Scheme 1). During the expansion process, three different stages are observed. In stage-I, natural graphite will be oxidized using potassium permanganate (KMnO_4_), HNO_3_, H_2_O_2_, *etc.*^92^ In this oxidation process, the molecules and ions of sulphuric acid (H_2_SO_4_) or nitric acid (HNO_3_) were inserted into the layers of the graphite crystal lattice and is known as the intercalation process. The resultant material is called oxidized graphite. In stage-II, water will be used to remove the excess intercalant ions/compounds by the washing process and then, the sample will be dried to maintain its physical properties such as malleability, microstructure, castability, *etc.* In the final stage-III, the oxidized graphite will be heated in a high-temperature reactor to achieve the thermally expanded graphite. When the sample is treated at >600 °C, the expansion of graphite layers takes place due to gaseous products formed by the decomposition of H_2_SO_4_ or HNO_3_, which helped to separate the adjacent graphite layers. Due to this expansion process, the interlayer distance (from 0.334 nm) between adjacent graphite layers can be increased up to 300 times.^93^

**Fig. 5:**
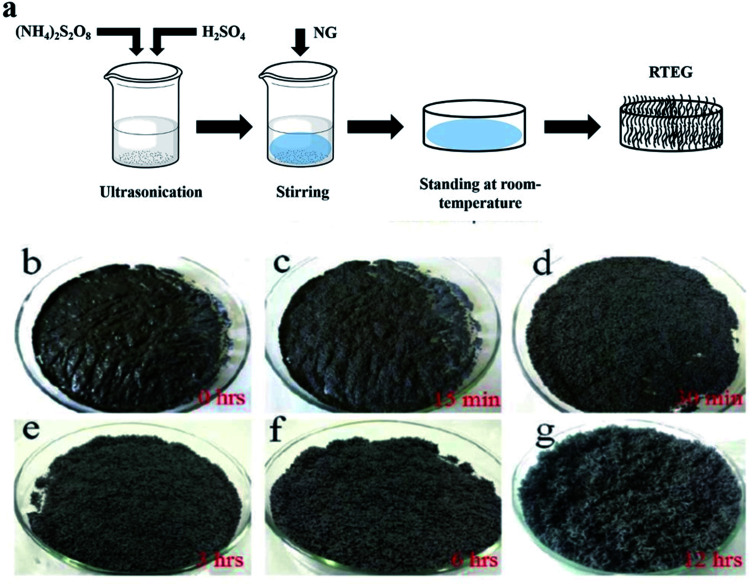
(a) The schematic diagram of TEG preparation by the one-step room-temperature method. (b–g) Photographic images of the graphite intercalation and morphological evolutions with time from 0 to 12 h.^94^ Adapted with permission from ref. 94 copyright 2017 Elsevier.

Liu *et al.*^94^ synthesized TEG by a one-step room-temperature method which showed an expansion volume up to 225 times. This experiment was carried out using a binary-component system made of ammonium persulfate and concentrated H_2_SO_4_. It was reported that the usage of less quantity of H_2_SO_4_ resulted in a worm-like structure by controlled stirring and standing procedures. Hence, this method was found to be efficient to prepare TEG because it required low energy and generated less pollutants. During the TEG preparation, various morphological changes were observed, as shown in Fig. 5(a–g).

Çalin *et al.*^89^ studied the relationship between the porous structure and the expansion ratio of TEG by varying the expansion circumstances of graphite with various flake sizes. They found that the graphite flake size had an effect on the expansion ratio of TEG. For example, when graphite flakes with a +325 mesh (>44 μm) size range were used to prepare TEG, they obtained a low intercalation rate because of the small size of the flakes which led to a poor expansion ratio. Similarly, when TEG was prepared using graphite flakes of −10 mesh (<2 mm) size, they observed quite higher expansion ratios at all the temperatures (700, 900, and 1100 °C). The graphite flakes with −10 mesh size showed a maximum expansion ratio (80 ml g^−1^) at 1150 °C. TEG with an expansion ratio of ∼400 ml g^−1^ was achieved at 1150 °C using commercial expandable graphite (with +50 mesh, >300 μm) in a closed lid crucible (Table 2).

**Table 2:** Comparison of the expansion ratio of TEG prepared by using sulfuric acid and graphite flakes with various mesh sizes^89^

S. No.	Mesh size and flake thickness of graphite	Expansion ratio and temperature	Expansion ability
1	−10 mesh (<2 mm)	800 ml g^−1^ at 1150 °C	Highest expansion rate
2	+50 mesh (>300 μm)	400 ml g^−1^ at 1150 °C	High pore volume and a moderate expansion rate
3	+325 mesh (>44 μm)	14 ml g^−1^ at 800 °C	Poor intercalation and a low expansion rate

The closed crucible helped the graphite flakes from burning on the surface and their escape during the expansion process. The maximum pore volume was achieved for TEG obtained from +50 mesh expandable graphite than −10 mesh which was confirmed by scanning electron microscopy analysis. The influence of the graphite particle size was also studied by Dhakate *et al.*^95^ for TEG based composite bipolar plates in polymer electrolyte membrane fuel cells. Four types of graphite flakes were taken (30, 50, 150 and 300 μm) and used for the fabrication of composite based bipolar plates with phenolic resin as a polymer matrix. The electrical conductivity of the bipolar plates was 150 S cm^−1^, and was found to be high for 300 μm sized graphite flakes. However, 30 μm sized graphite flake based composite plates showed high mechanical properties and low electrical conductivity.^95^ The above study indicated that the expansion ratio of natural graphite flakes depended on their particle sizes. Also, Wang *et al.* prepared TEG using graphite flakes with different mesh sizes (80, 120, 150 and 200) and found that the expansion ratios increased with the mesh size.^45^

In a similar manner, TEG was prepared by using a microwave irradiation method which provided TEG with uniform expansion of graphite layers.^96^ It was considered as one of the effective methods for the preparation of TEG. Van Pham *et al.*^97^ obtained TEG through a two-step process. First, graphite flakes, KMnO_4_, acetic anhydride, and perchloric acid were mixed in a ratio of 1 : 0.5 : 1 : 0.4 (g g^−1^) for a few seconds and the mixture was kept in a microwave oven at 360 W for 50 s to achieve the expansion of graphite sheets. Wei *et al.*^98^ also synthesized TEG using natural graphite with HNO_3_ and KMnO_4_ as oxidizing agents. The mixture was stirred at room temperature for 3 min. After that, the mixture was kept in a microwave oven and irradiated for 60 s at a power rate of 700 W. At this time, exfoliation of the graphite layers was observed with fuming and lightening effects. The worm-like structure of TEG was observed by scanning electron microscopy (SEM) analysis and the surface morphology of TEG exhibited a well exfoliated structure where expansion occurred along the direction of the *c*-axis.

### 2.1 Role of TEG in nanocomposites

**Fig. 6:**
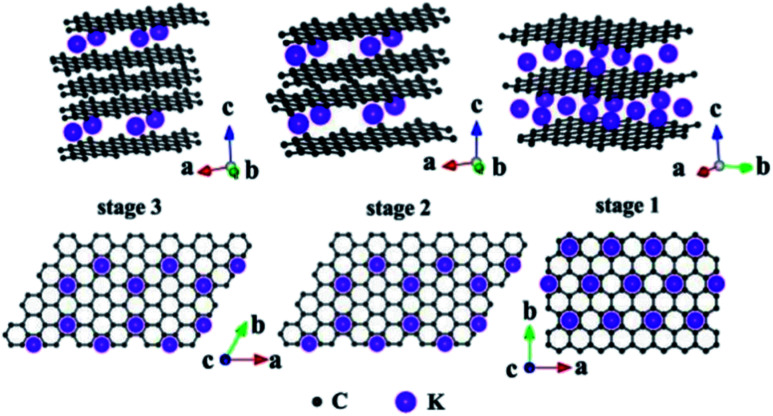
Structural illustrations of potassium based – GICs;^102^ side view and top view adapted with permission from ref. 102 copyright 2020 Elsevier.

Due to the presence of multiple pores and functional groups such as –COOH and –OH on the TEG surface, it exhibited high physical and chemical properties compared to graphite.^99^ For instance, Zheng *et al.*^100^ prepared polymethyl methacrylate and TEG based composites which showed high mechanical (storage modulus of 3.4 GPa and loss modulus of 380 MPa) and electrical (10^−4^ S cm^−1^) properties. This was due to the high surface area and conductive nature of TEG which helped to enhance the above properties. During the synthesis of TEG, strong oxidizing agents such as KMnO_4_, HNO_3_, H_2_SO_4_, hydrogen peroxide (H_2_O_2_) and other intercalating agents (halogens, alkali metals, *etc.*) have been used. These agents created several oxygen-rich functional groups on the surface of TEG. As a consequence, the resulting TEG with these functional groups with a porous structure helped to interact with various molecules to form the composite materials.^45^ In particular, TEG with mesopores (2–50 nm) and macropores (>50 nm) was preferred to decorate with various molecules/compounds.^89,101^ Furthermore, due to the unique morphology of TEG, other molecules/ions/compounds/metals could be easily positioned within the open pores, nano-cavities or at the edges of graphene sheets of TEG which helped to form a uniform distribution of the supporting materials (Fig. 6).^89^ By tuning the temperature, duration of thermal treatment, selection of graphite particles with an optimum size and nature of intercalants, the expansion ratio, number of pores, and pore sizes of TEG can be controlled during the synthesis^91^ based on the requirements for the relevant applications.

**Fig. 7:**
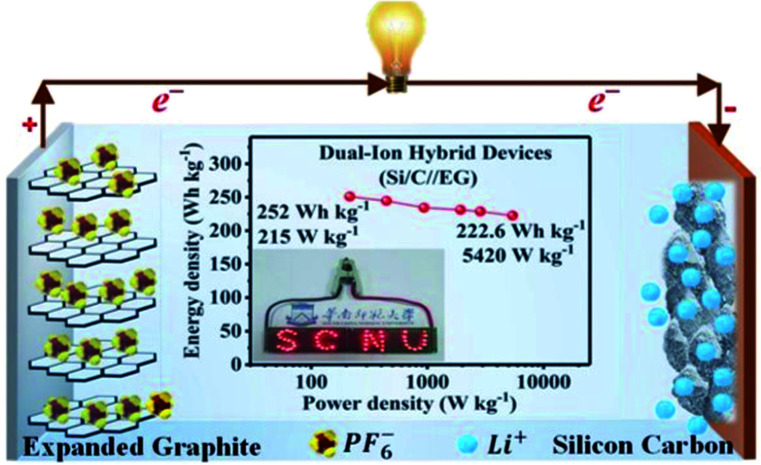
Schematic representation of the dual-ion hybrid devices^117^ fabricated with a silicon–carbon anode and expanded graphite cathode adapted with permission from ref. 117 copyright 2020 Royal Society of Chemistry.

Several studies have been reported on the thermal conductivity and electrochemical properties of TEG.^103,104^ TEG has also been used in the preparation of electrochemical sensors,^105^ fuel cells,^106,107^ batteries^108,109^ and supercapacitors.^110,111^ In particular, TEG was demonstrated in lithium-ion batteries (LIBs) with good cycling stability up to 100 cycles, and the first reversible capacity was 401 mA h g^−1^.^112^ Herein, the stacking arrangement of graphene sheets enhanced the lithium-ion transportation and the electron conductivity of the electrode. After 50 cycles, the obtained specific capacity was 381 mA h g^−1^ and the retention rate reached up to 95%. The interlayer spacing of intercalated TEG was further enhanced by the amorphous carbon-coated aluminum metal nanoparticles, thus forming a metal network structure and also played a vital role in the process of lithium-ion transportation by increasing the specific capacity and cycling stability. Huang *et al.*^113^ used TEG as a flexible conductive matrix to support the aluminum particles which were coated with carbon. The 3D network helped in the lithium ion transportation over larger distances and the agglomeration of aluminum was restrained. This anode material showed 75.9% capacity retention after fifty cycles. Hu *et al.*^114^ used intercalated carbon-coated trilithium vanadate (Li_3_VO_4_) nanoparticles with TEG as a loading carrier to support the fast diffusion of electrons, in which they have demonstrated that TEG could be used as a conductive material with enhanced electrical conductivity. Additionally, a self-standing flexible electrode was made using TEG to implant electrochemically active compounds such as silicon or sulfur for binder-free Li-ion batteries.^115^ Bai *et al.*^116^ studied the electrochemical properties of graphene sheets, TEG and natural graphite as anode materials for lithium-ion batteries. The large voltage hysteresis resulted for graphene sheets and expanded graphite electrodes due to the surface functional groups and crystalline defects. The kinetic properties of the graphene sheets, TEG and natural graphite electrode were studied by AC impedance measurements. The graphene sheets and TEG showed appreciable cycling stability with 90–95% of coulombic efficiency after the first cycle. The obtained reversible capacities of graphene sheets were 1130 and 636 mA h g^−1^ at a current density of 0.2 and 1 mA cm^−2^ which was higher than that of TEG and natural graphite. He *et al.*^117^ designed a dual-ion hybrid energy storage system using TEG as an anion-intercalation supercapacitor-type cathode and graphite/nanosilicon@carbon (Si/C) as a cation intercalation battery-type anode for effective energy storage application (Fig. 7). Herein, the TEG cathode stores the energy through electrochemical double layer capacitance using the unique faradaic pseudocapacitive negative anion intercalation behavior. The Si/C//EG device exhibited energy density in the range of 252–222.6 W h kg^−1^ at a power density of 215 to 5240 W kg^−1^, which is the highest reported value for hybrid systems. This hybrid dual ion battery exhibited an enhanced capacity retention around 95% after 1000 cycles, which was higher than that of the commercially available supercapacitors,^118^ lithium-ion capacitors and commercial lithium-ion batteries.^117^

## 3. Applications of TEG

### 3.1 TEG–metal based composites for energy applications

**Fig. 8:**
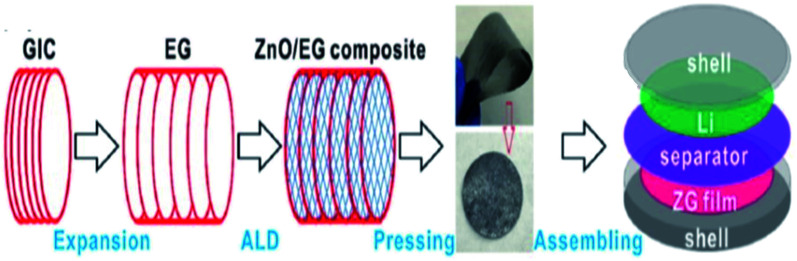
Schematic diagram of the preparation of the ZnO/TEG composite preparation as a binder-free anode for lithium-ion batteries^115^ which was adapted with permission from ref. 115, copyright 2017 American Chemical Society.

Zhao *et al.*^112^ synthesized composite materials made of carbon-coated aluminium/TEG by chemical vapor deposition and ball milling methods. They studied the mechanism of aluminium intercalation and deintercalation in the composite. It was found that the intercalation process of TEG further increased the interlayer spacing between the layers and formed a metallic aluminium network structure. This composite showed high lattice intercalation sites with an aluminium doping of 30%. In addition, it was used as the anode material for lithium-ion batteries which showed high cycling stability with a specific capacity of 381 mA h g^−1^ after 50 cycles and the retention rate performance was up to 95%.^112^ Recently, He *et al.*^111^ stated that a polymer binder was used for the attachment of the catalyst dissolved substance onto the surface of a glassy carbon electrode. But, it did not increase the series resistance as well as masked the catalytically active sites, which could affect the electrochemical properties. To overcome this barrier, a composite was prepared by combining TEG, acetylene black and polytetrafluoroethylene emulsion in a tablet form called a TEG matrix. Later, molybdenum disulphide (MoS_2_) was grown on it by hydrothermal synthesis. The activity of the hydrogen evolution reaction (HER) of the composite was increased due to the presence of the TEG matrix as a conductive substrate which had a charge transfer resistance of about 0.919 Ω cm^2^, overpotential of about 230 mV (*j* = 10 mA cm^−2^) and Tafel slope of 77 mV dec^−1^. Chen *et al.*^1^ synthesized PtCo nanoparticles on TEG composites by a solution-phase reduction method with ethylene glycol for direct methanol fuel cells. The electro-catalytic performance was studied by cyclic voltammetry and chronoamperometry.^1^ The PtCo/TEG based composite showed high electrocatalytic activity and long-term stability because of TEG. It had exceptional electrochemical stability in acidic medium and proved to be a favorable anode material. The PtCo supported TEG showed a larger electrochemical active surface area which was higher (55.75 m^2^ g^−1^) than that of Pt/C and PtCo/MWCNTs (multiwall carbon nanotubes). Also, the mass specific current (*I*_f_) of PtCo/TEG (525.08 mA mg^−1^) was high, when compared with that of Pt/C (320.75 mA mg^−1^) and PtCo/MWCNTs (474.59 mA mg^−1^). Therefore, the authors concluded that the PtCo/TEG composite exhibited better electrocatalytic performance and reduced the accumulation of oxidized carbonaceous species on the surface of the catalyst. Li *et al.* demonstrated a binder-free anode for lithium-ion batteries by using zinc oxide and TEG. They used atomic layer deposition to prepare ZnO from dimethyl-zinc (source) and water as the oxidant. The presence of TEG in the composite provided a conductive channel and mechanical support for the ZnO membrane. This composite was also prepared as a flexible film without a binder to act as an anode in the setup^115^ (Fig. 8).

**Fig. 9:**
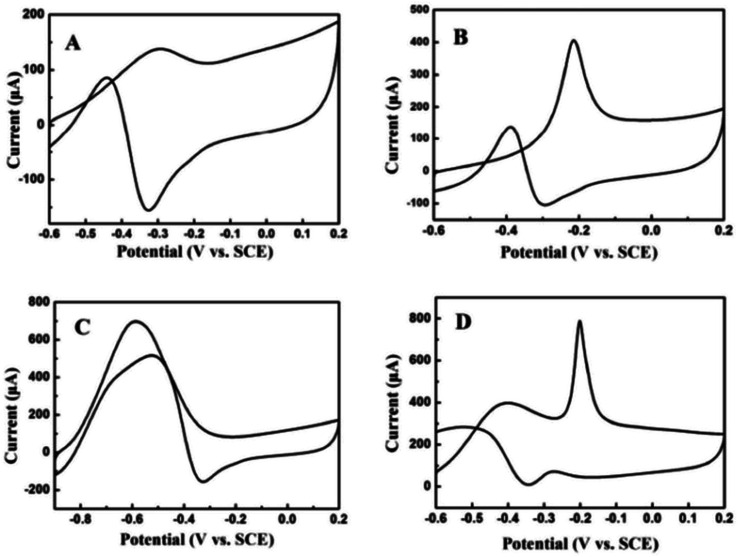
Cyclic voltammograms recorded for 0.05 M (A) ethanol, (B) ethylene glycol, (C) formic acid and (D) formaldehyde^31^ in 1.0 M KOH on the PdNPs/TEG–MWCNT composite modified glassy carbon electrode at a scan rate of 100 mV s^−1^. Adapted with permission from ref. 31, copyright 2018 Elsevier.

The electrocatalytic oxidation of different liquid fuels (ethanol, ethylene glycol, formic acid, methanol, *etc.*) was studied using the electrocatalyst of palladium nanoparticles (PdNPs) deposited on TEG–MWCNTs.^31^ The rate of the oxidation process of these liquid fuels on the composite has been reported in the increasing order of aldehyde: (formaldehyde) > carboxylic acid (formic acid) > alcohols (ethylene glycol > ethanol > methanol) (Fig. 9). The morphology of the composite was studied using SEM where TEG exhibited the wrinkled layers along with the pores that usually offer a large surface area and more active sites on the surface. When comparing the MWCNT film with the TEG–MWCNTs, the aggregations of MWCNTs were much reduced. Also, the surface of the TEG–MWCNTs turned out to be relatively rough. Such a surface was favorable for the electro-deposition of the PdNP catalyst. The PdNPs were spherical in shape with an average size of 5 nm which was embedded in the TEG–MWCNTs.^31^

Wen *et al.*^119^ fabricated a TiP_2_O_7_/TEG-based nanocomposite as an anode by the sol–gel method for lithium-ion batteries. The introduction of TEG into the composite increased the conductivity and reduced the particle size of TiP_2_O_7_ (50–100 nm). The effect of the various ratios of TEG in the composite was studied, and the optimized ratio was 30%. Next, the electrochemical performance of the TiP_2_O_7_/TEG-based nanocomposite was studied in both organic and aqueous electrolytes. The TiP_2_O_7_/TEG electrode showed a reversible capacity of 66 mA h g^−1^ at 0.1 A g^−1^ with a suitable potential of −0.6 V using aqueous electrolytes. An enhanced cycling stability was observed with 75% capacity retention after 1000 cycles at a current density of 0.5 A g^−1^. The TiP_2_O_7_/TEG anode was used in organic electrolyte (1 M lithium sulfate, Li_2_SO_4_) where LiMn_2_O_4_ as the cathode showed a specific energy of 60 W h kg^−1^ with a voltage of 1.4 V. It also showed higher rate capability and outstanding cycling performance with a capacity retention of 66% over 500 cycles at 0.2 A g^−1^ and 61% at 1 A g^−1^ over 2000 cycles.

### 3.2 Applications of TEG in sensors

**Fig. 10:**
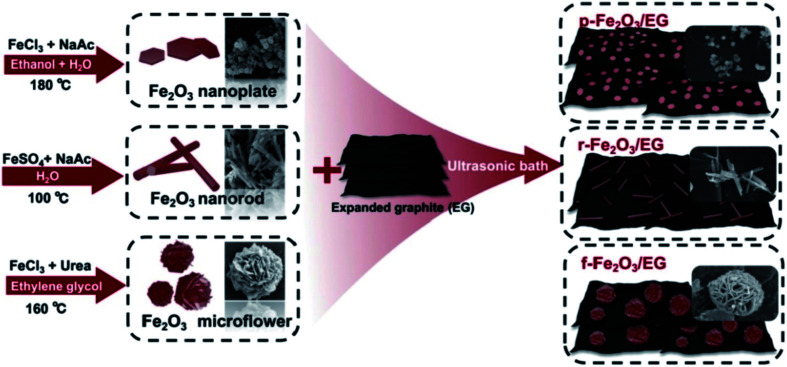
Different fabrication steps used for the preparation of iron oxide nanomaterials with three different morphologies (nanoplates, nanorods, and 3D flower-like structures)^121^ with TEG as composites. Adapted with permission from ref. 121 copyright 2019 Elsevier.

The TEG/silver-zeolite-epoxy composite modified electrode showed good electro-catalytic activity for urea oxidation in alkaline solution and linearly responded for the detection of urea in the range of 0.4 to 2.8 mM with a limit of detection (LOD) of 0.05 mM.^120^ Chen *et al.*^121^ synthesized iron oxide nanomaterials with three different morphologies such as nanoplates, nanorods and 3D flower-like structures which were then coupled with TEG (Fig. 10).

These composite modified glassy carbon electrodes (GCE) were used for the electrochemical detection of three important organic pollutants. Among these, 3D flower-like iron oxide/TEG showed the best electrocatalytic performance and was employed for highly selective and sensitive detection of tetrabromobisphenol A (LOD = 1.23 nM), sunset yellow (LOD = 0.89 nM) and tartrazine (LOD = 2.17 nM).

In a recent study, cerium oxide (CeO_2_) was synthesized in three different morphologies (nanocubes, nanopolyhedra, and nanorods). After that, each one of these structures was individually mixed with TEG to prepare nanocomposites.^122^ Compared to the other two morphologies, nanorod-CeO_2_ exhibited the best electrochemical sensing performance towards Cd (LOD = 0.39 μg l^−1^) and Pb ions (LOD = 0.21 μg l^−1^) because of the high surface area and the lower charge transfer resistance. Similarly, PdO nanoparticle decorated TEG was also prepared and coated on the GCE for the electrochemical sensing of tetrabromobisphenol A (LOD = 1.3 nM), hydroquinone (LOD = 26 nM) and catechol (LOD = 17 nM) at nanomolar levels.^4^ The large surface area and more surface-active sites of TEG made it a perfect material for the construction of nanocomposites to prepare electrochemical sensors with high sensitivity, selectivity and reproducibility.

### 3.3 TEG–polymer composite materials

The TEG/epoxy composite was synthesized by using the solution mixing method, and the interaction between the TEG and epoxy matrix was examined^123^. The electrical, mechanical, and thermal properties of the resin were enhanced by the introduction of TEG into the nanocomposite. TEG sheets played a major role in decreasing the strain and increasing the tensile strength of the composite compared to epoxy resin. Furthermore, upon the addition of 9 wt% of TEG to the epoxy, the electrical conductivity increased from 10^−15^ to 10^−5^ S cm^−1^, also the thermal properties of the composite increased from 340 to 480 °C and the mechanical properties were found to be increased by more than 30%. These enhancements were credited to the good dispersion of TEG in the epoxy matrix.

Zheng *et al.*^124^ reported a conductive polymer composite made of poly[styrene-*co*-acrylonitrile] (PSAN) and TEG. They reported that the oxidation and expansion conditions for TEG synthesis were more important and must be carefully controlled to avoid excessive oxidation of TEG which may led to a low expansion ratio and a honeycomb structure. The *in situ* polymerization of the styrene and acrylonitrile monomers inside the diamond-shaped pores of TEG resulted in the PSAN/TEG composite with a graphite network throughout the entire polymer matrix. In particular, the in-plane (surface) electrical resistivity was found to be 8.5 × 10^−3^ Ω cm and the through-thickness (between upper and bottom surfaces) resitsivity was found to be 1.2 × 10^−2^ Ω cm. The improved electrical conductivity of the PSAN/TEG composite was credited to the graphite ligaments through the entire composite sheet which formed a unique graphite network^124^.

Reticulated vitreous carbon (RVC) composite foam was prepared using TEG as a filler.^40^ When carbon foam derived from polymers as a precursor exhibited brittleness, some reinforcement materials were added to improve its mechanical properties. In this case, the brittle nature of the vitreous carbon composite was improved and the compressive strength was improved from 0.57 MPa to 0.72 MPa by the addition of TEG. Poly furfuryl alcohol was mixed with the carbon matrix and the wt% of TEG was varied in the resin to fill the polyurethane foam template. Due to the presence of TEG, the reticulated vitreous carbon composite did not form any cracks after the heat treatment up to 100 °C and the compression strength increased along with the electrochemical surface area. Also, TEG had increased the interaction with the matrix and acted as a barrier for the crack propagations. This micro-structure improvement was observed for the wt. ratio of 1% TEG. The crystallinity of the RVC–TEG composite was improved due to the addition of TEG which was also helped to enhance the charge transfer reaction and specific electrochemical surface area of reticulated vitreous carbon electrodes. Yi *et al.*^125^ reported an anode (3D) material containing stannic oxide, polyaniline and TEG by solvothermal and oxidative polymerization methods. The electrochemical performance of the composite was enhanced by the synergistic effects between TEG and polyaniline since the polyaniline can stabilize the structure, improve ionic diffusion during the charge–discharge process and reduce the electric contact resistance of the electrode materials with the addition of the electrolyte. The 3D TEG/tin oxide/polyaniline (TEG@SnO_2_@PANI) showed a coulombic efficiency of 77.8%, initial reversible capacity of 1021 mA h g^−1^ and after 100 cycles, the value was maintained at 408 mA h g^−1^. Wang *et al.*^126^ prepared a polypyrrole/TEG nanocomposite by the vacuum-assisted intercalation method *via* the *in situ* oxidation polymerization process without a surfactant. The pyrrole was strongly adhered on both sides of the TEG nanosheets and then polypyrrole was coated on TEG by the polymerization reaction. In this work, TEG nanosheets played an important role as current collectors during the charging and discharging process by speeding up the electron transport. In addition, TEG in the composite worked as a self-supporting skeleton and prevented the volumetric swelling and shrinking of the composite for high cycling stability. The polypyrrole/TEG nanocomposite showed a specific capacitance of 86.1% even after 2000 charge and discharge cycles when 10% weight ratio of TEG was added.

In addition, conducting polymer composites with various morphologies can be synthesized for various applications such as fibers, spheres, sheets, bulk, wires, *etc.*^127^ For example, composites with porous nature could incorporate several chemical functionalities into the open site of the pores which increased the surface area, provided more electrochemically active sites and a good electrolyte–electrode interface for fast ion transport to improve the specific capacity or catalytic activity.^128^ If we compare the aspect ratio, the wire and rod-shaped polymer composites exhibited a high aspect ratio compared to the sphere shaped polymer composite. Therefore, the transport of electrical carriers along one controllable direction was possible.^129^ The wire shaped composite materials showed high electronic conductivity and optical properties due to their high aspect ratio.^128^ Furthermore, nanosheets with a nanoporous structure could increase the molecular interactions due to the increased specific surface area.^128^ Also, polymer composites with sheet and fiber like morphologies could offer high flexibility than the bulk phase structured composite which was used in flexible free-standing electrodes. It is worth mentioning that conducting polymer composites show more advantages such as light weight, high flexibility, stretchability and easy processing to manufacture soft robotic skin, artificial muscles, human–machine interfaces, *etc.*^130^ As discussed above, we believe that TEG based polymer composites can be prepared with enhanced mechanical, electrical and electrochemical properties.

### 3.4 TEG and enzyme based biocomposite electrodes

Ramesh *et al.*^131^ constructed a sol–gel-based electrode using TEG modified with dopamine by covalent attachment through amide linkages *via* –COOH groups present on TEG. For this, TEG was dispersed in organically modified silicates to prepare the sol–gel. The as-prepared TEG/dopamine modified electrode showed high electrocatalytic activity towards the oxidation of nicotinamide adenine dinucleotide reduced form (NADH). The TEG/dopamine modified electrode was used to detect ethanol in the presence of nicotinamide adenine dinucleotide oxidized form (NAD^+^) and alcohol dehydrogenase. This TEG/dopamine composite was prepared without using any binder and recompressed in a compact pellet for use as an electrode.

**Fig. 11:**
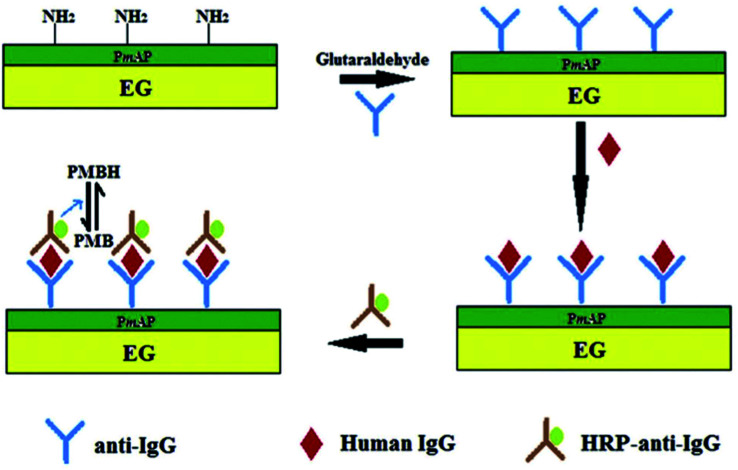
Schematic representation of the sandwich-type fabrication of the electrochemical immunosensor to detect human IgG.^136^ Adapted with permission from ref. 136, copyright 2013 Elsevier.

A low-cost electrode was designed for sensing hydrogen peroxide (H_2_O_2_) and glucose using a stamped multilayer lamination process.^132^ For this work, TEG was synthesized with full expansion and thus it was possible to fabricate hundreds of electrodes by the stamping method.^133–135^ Also, this method could be used to prepare electrodes on flexible substrates such as paper and plastic which are more economical and useful for sensing applications. Interestingly, researchers have constructed inexpensive electrodes that were laminated on a 100% biodegradable cellulose substrate which can be disposed easily without any toxic effects on the environment. To detect glucose, the laminated electrodes were treated with glucose oxidase which exhibited a linear response towards H_2_O_2_ (from 1.95 to 500 μM) with a LOD of 1.91 μM. This sensor detected glucose in the range of 0 to 2.5 mM with a LOD of 1.22 μM in phosphate buffer solution (PBS.) It is worth pointing out that this electrode did not respond to higher levels of glucose in blood.^132^ Therefore, these laminated electrodes may be useful for exploration purposes and non-invasive detection of glucose at low concentrations in sweat, tears and saliva. In addition, the endogenous peroxide sensing abilities of untreated electrodes could be useful for peroxide sensing, glucose-based fuel cells and detection of analytes at low concentrations. Tao *et al.*^136^ fabricated an electrochemical immunosensor based on poly(*m*-aminophenol) immobilized with the anti-human immunoglobulin G (IgG) antibody and TEG (Fig. 11). Herein, glutaraldehyde was used as a coupling agent where the two aldehyde groups were found to react with the amino groups of poly(*m*-aminophenol) which was later deposited on the TEG surface through the amino group of the antibody. In this immunoassay method, the target antigen was a human IgG, the probing antibody was horseradish peroxidase coupled with goat anti-human (IgG) and poly methylene blue (PMB) was the electron transfer mediator. The electrochemical measurements showed that the oxidation peak current of PMBH (reduced state of PMB) was proportional to the concentrations of IgG from 5–60 μg ml^−1^ and the LOD was found to be 0.19 μg ml^−1^.^136^

## 4. Challenges and future prospects

During the preparation of TEG by using natural graphite *via* acid treatment and the metal intercalation process, various functional groups (–OH, –COOH, *etc.*) were generated on the surface of TEG.^99^ In addition to high temperature, metal intercalates and graphite with different sizes are required to synthesize TEG.^137^ However, this method could generate more acid wastes, reagents and also the expansion ratio of the graphite layer was uncontrollable during the synthesis process. Sometimes, TEG became an insulator due to the higher degree of oxidation due to which the oxygen content increased on the TEG material after the synthesis. This is one of the limiting factors of TEG. In addition, the lateral dimensions of TEG are larger and thick when compared to those of the graphene platelets. Therefore, the electrochemical interface will be affected during the reaction.^4^ It is important to point out that a high temperature is required for the expansion process of TEG which may also cause safety issues and more precautions should be taken. Also, synthesis of TEG with a good expansion ratio and high yield is still a challenge because of the above-mentioned factors. On the other hand, the removal of intercalate ions from TEG is also required. The presence of trace level metals in the final product may either have positive or negative effects during the electrochemical applications in supercapacitors, batteries, fuel cells, sensors, *etc.* Moreover, in order to disperse TEG in a suitable surfactant or solvent, additional instruments such as an ultra-sonicator were required to obtain stable dispersion along with composite materials. These are some of the challenges that have to be overcome in order to use TEG effectively in various commercial applications without any limitations.

TEG had also been used widely as a phase-changing material,^66,138^ fire retardant,^139,140^*etc.* due to its excellent thermal stability. Compared to graphene and CNTs, TEG is less expensive and easy to prepare. However, the electrochemical applications of TEG in the energy storage applications, flexible sensors, hydrogen evolution reaction (HER), oxygen evolution reaction (OER), and water purification process^97,141,142^ must be further explored in detail and potential real-world applications should be demonstrated.

For this purpose, new methods and techniques should be developed to demonstrate TEG in the fabrication of electrochemical energy storage devices (batteries, supercapacitors and fuel cells) and sensors. We believe that extensive research on TEG will enable us to compare the advantages and disadvantages of TEG with CNTs and other graphene-based electrodes in terms of their electrochemical performances. In recent years, flexible electronics and electrodes have received more attention.^143^ In this direction, the future aspects of TEG and its suitability for the development of flexible^108,144^ (asymmetric supercapacitor, nanoelectronics, *etc.*) and wearable electronic devices for health care monitoring will be more promising and useful for healthcare applications. Due to its anti-corrosion properties, TEG can be used as an inhibitor in the painting of ships and can also be used in the manufacturing of smart sensor devices for checking the quality of food and reporting the moisture content of the packaged products. As we pointed out in this review, TEG has a lot of potential to be used in the nanocomposite preparation for various electrochemical applications.

## 5. Conclusion

Carbon materials have been used in various applications because of their easy availability with various microstructures. The key factors that should be considered during the fabrication of electrodes include surface area, interconnected pore structure, controlled pore size matching to the intercalates, high electrical conductivity, the presence of functional groups, and high thermal and electrochemical stability. All these requirements can be fulfilled by employing TEG to prepare the composite material for electrochemical applications. TEG can be prepared by two common methods such as high temperature expansion after acid treatment or microwave radiation in the presence of intercalants and oxidizing agents. Its distinct sandwich structure and elongated distance between the graphene layers provided an opportunity to intercalate with ions or molecules between the layers. At high temperatures, intercalates helped in the expansion of graphite layers by thermal expansion due to the gas evolution process. The force produced by the exhaust of these gaseous molecules started the exfoliation process of graphite and therefore a significant growth in volume occurs; TEG with a very low density was produced. The surface morphology and pore structure of TEG can be tuned by the applied temperature and selection of suitable graphite flake sizes. It was possible to use TEG in the fabrication of electrode materials with high efficiency, high stability and high reliability as demonstrated in other publications. Finally, TEG proved to be an excellent composite material for various electrochemical applications. In many of the nanocomposites, we were able to find that TEG was used in order to avoid binders in the energy storage applications. Also, TEG acted as a load carrier that supported the fast diffusion of electrons and thereby it was used as a conductive material to improve the electrical conductivity. The crumpled layers along with the porous nature of TEG might offer a large surface area thereby providing more active sites on the surface which could be helpful for electrochemical sensors. It was also demonstrated that TEG-based composites could be used to develop electrochemical sensors and biosensors with high selectivity and sensitivity for various analytes.

## Conflicts of interest

The authors declare that they have no known competing financial interests or personal relationships that could have appeared to influence the work reported in this paper.

## Supplementary Material
